# Comment on “Radiological risk factors in different patterns of patellar instability”

**DOI:** 10.1186/s43019-026-00324-6

**Published:** 2026-08-11

**Authors:** Eşref Selçuk

**Affiliations:** Department of Orthopaedics and Traumatology, Trakya University, Edirne, Turkey

**Keywords:** Patellar instability, Patellar dislocation, Tibial tubercle–trochlear groove distance, Trochlear dysplasia

## Abstract

I read with great interest the recently published article by Chouhan et al. examining radiological risk factors across different patterns of patellar instability. The authors investigated trochlear dysplasia, tibial tubercle–trochlear groove (TT–TG) distance, and patellar height across single-episode, recurrent, and habitual patellar dislocation phenotypes. While this phenotype-based imaging approach addresses a clinically relevant question, several methodological and conceptual considerations may limit the robustness and clinical translatability of the reported between-group differences. The analysis was performed at the knee level despite inclusion of bilateral cases, without accounting for within-patient clustering, potentially inflating statistical significance. In addition, exclusion of patients who underwent stabilization procedures, particularly among single-episode dislocators, may introduce spectrum and selection bias, exaggerating apparent phenotype contrasts. The absence of formal inter- and intraobserver reliability assessment further constrains interpretation, especially given the known measurement variability of TT–TG distance, patellar height indices, and dysplasia grading. Reported TT–TG differences between single-episode and recurrent instability were small, with substantial overlap and without confidence intervals, reliability metrics, or threshold-based analyses, limiting clinical actionability within contemporary multifactorial decision frameworks. Inclusion of habitual patellar dislocation, often a developmental condition with distinct and entrenched structural abnormalities, within the same comparative construct as episodic instability raises concerns regarding construct validity and extrapolation of management implications. In conclusion, this work tackles an important clinical topic, and its conclusions could be further consolidated by incorporating cluster-aware analyses, clearly documenting exclusion patterns and reporting measurement reproducibility and TT–TG analyses aligned with clinically actionable thresholds within a multifactorial anatomical framework.

 I read with interest the article by Chouhan et al., “Radiological risk factors in patellar instability: a comparative analysis of single-episode, recurrent, and habitual patellar dislocation,” which examines trochlear dysplasia, TT–TG distance, and patellar height (Caton–Deschamps index) across distinct clinical phenotypes [1]. A phenotype-based imaging comparison addresses a clinically relevant question; however, several design and analytical considerations limit the strength and clinical translatability of the reported between-group differences.

First, the analysis is performed at the knee level (124 knees from 106 patients), meaning that in patients with bilateral involvement, the two knees are not fully independent observations. However, the main comparisons are based on simple chi-squared tests, which assume independence between observations. When both knees from the same patient are included, this assumption may be violated, and the results may appear more statistically significant than they truly are [2]. Performing a sensitivity analysis using only one knee per patient, or applying analytical approaches that account for patient-level clustering, would strengthen the robustness and credibility of the main conclusions.

Second, spectrum/selection effects may exaggerate phenotype contrasts. Excluding patients who underwent stabilization procedures, particularly within the single-episode group, risks preferentially removing higher-risk first-time dislocators and retaining milder cases, creating an artificial severity gradient [3]. Transparent accounting of exclusions by phenotype (including how many single-episode cases were excluded for operative management and whether their imaging features differed) is essential to interpret the comparative framing.

Third, although the authors acknowledge in the Limitations section that inter- and intraobserver variability was not assessed, the reliance on consensus readings does not replace formal reproducibility metrics. Given the well-documented sensitivity of TT–TG distance and patellar height indices to imaging technique, patient positioning, and slice selection, and the recognized variability in dysplasia grading, reporting observer agreement (intraclass correlation coefficients for continuous measures and kappa statistics for categorical assessments) remains important [4]. This is particularly relevant when the observed between-group differences are modest and form the basis of the study’s comparative interpretations.

TT–TG is presented in a way that limits discriminability and decision relevance (Table 2). Table 2 illustrates that mean TT–TG differs by only ~2 mm between single-episode and recurrent instability (11.69 ± 3.6 versus 13.81 ± 2.64 mm), with substantial overlap in dispersion. Without confidence intervals, quantified reliability (particularly for magnetic resonance imaging [MRI]-based TT–TG), and cluster-robust inference, it is difficult to know whether this small separation reflects a true phenotypic signal or measurement variability. Moreover, contemporary “menu à la carte” concepts emphasize that TT–TG should be interpreted as one component of a multifactorial anatomical construct, with clinical utility driven less by group means than by threshold exceedance and interaction with major co-factors (e.g., trochlear dysplasia and patella alta). In other words, without threshold-exceedance reporting and reproducibility metrics, the TT–TG comparison risks being statistically describable yet clinically nonactionable [5]. Accordingly, reporting effect sizes with 95% confidence intervals (CIs) and the proportion of knees exceeding clinically used thresholds (e.g., > 15 mm and > 20 mm) would provide a more interpretable and decision-relevant comparison than means alone. The markedly larger standard deviation (SD) in the habitual group (19.37 ± 8.52 mm) further suggests heterogeneity and/or limited precision, reinforcing the need for careful interpretation.

Lastly, construct validity merits clearer framing. Habitual patellar dislocation is frequently developmental with entrenched, often multiplanar structural abnormalities, whereas single-episode/recurrent instability more commonly represents episodic disease in which imaging variables function as risk modifiers. Analyzing habitual dislocation within the same comparative framework risks quasi-tautological interpretation and may invite inappropriate extrapolation of cut-points or management implications from episodic instability to a fundamentally different condition [6, 7]. Treating habitual dislocation as a distinct etiologic subgroup, or providing sensitivity analyses excluding habitual cases, would strengthen conceptual validity and clinical translatability.

In conclusion, this work tackles an important clinical topic, and its conclusions could be further consolidated by incorporating cluster-aware analyses, clearly documenting exclusion patterns and reporting measurement reproducibility and TT–TG analyses aligned with clinically actionable thresholds within a multifactorial anatomical framework.

## Data Availability

No datasets were generated or analyzed during the preparation of this Letter to the Editor.
